# A High-Density SNP and SSR Consensus Map Reveals Segregation Distortion Regions in Wheat

**DOI:** 10.1155/2015/830618

**Published:** 2015-10-27

**Authors:** Chunlian Li, Guihua Bai, Shiaoman Chao, Zhonghua Wang

**Affiliations:** ^1^College of Agronomy, Northwest A&F University, Yangling, Shaanxi 712100, China; ^2^State Key Laboratory of Crop Stress Biology for Arid Areas, Northwest A&F University, Yangling, Shaanxi 712100, China; ^3^Agronomy Department, Kansas State University, Manhattan, KS 66506, USA; ^4^Hard Winter Wheat Genetics Research Unit, USDA-ARS, Manhattan, KS 66506, USA; ^5^Cereal Crops Research Unit, USDA-ARS, Fargo, ND 58102, USA

## Abstract

Segregation distortion is a widespread phenomenon in plant and animal genomes and significantly affects linkage map construction and identification of quantitative trait loci (QTLs). To study segregation distortion in wheat, a high-density consensus map was constructed using single nucleotide polymorphism (SNP) and simple sequence repeat (SSR) markers by merging two genetic maps developed from two recombinant-inbred line (RIL) populations, Ning7840 × Clark and Heyne × Lakin. Chromosome regions with obvious segregation distortion were identified in the map. A total of 3541 SNPs and 145 SSRs were mapped, and the map covered 3258.7 cM in genetic distance with an average interval of 0.88 cM. The number of markers that showed distorted segregation was 490 (18.5%) in the Ning7840 × Clark population and 225 (10.4%) in the Heyne × Lakin population. Most of the distorted markers (630) were mapped in the consensus map, which accounted for 17.1% of mapped markers. The majority of the distorted markers clustered in the segregation distortion regions (SDRs) on chromosomes 1B, 2A, 2B, 3A, 3B, 4B, 5A, 5B, 5D, 6B, 7A, and 7D. All of the markers in a given SDR skewed toward one of the parents, suggesting that gametophytic competition during zygote formation was most likely one of the causes for segregation distortion in the populations.

## 1. Introduction

Segregation distortion in some chromosome regions, in which the frequencies of segregating alleles skew from their expected Mendelian ratios, is a widespread phenomenon in plants and animals and is an important evolutionary force [1]. A segregation distortion region (SDR) can be identified by segregation distortion markers (SDMs) that significantly deviate from the expected Mendelian segregation. Segregation distortion loci (SDLs) or linked markers showing segregation distortion toward the same parent are often clustered in a small genomic region [2]. Segregation distortion can be caused by competition among gametes for preferential fertilization and by abortion of the male or female gametes or zygotes [1]. Segregation distortion can also occur as a result of conscious or unconscious selection during development of mapping populations. Segregation distortion affects accuracy of linkage map construction by introducing errors in map distance estimation and marker order and thus could affect mapping quantitative trait loci (QTLs) when many SDLs are present [3, 4]. Although SDLs may not have a large impact on the estimations of QTL position and effect in a large mapping population, the QTL detection power can be lower when QTLs and SDLs are closely linked [5]. Therefore, it is necessary to consider segregation distortion in map construction and QTL analysis.

Most interest in segregation distortion is currently centered on the genomic regions harboring markers with segregation distortion in different populations and their effects on linkage map construction and QTL mapping. Segregation distortion has been described in many crop species, including barley (*Hordeum vulgare*) [6], chickpea (*Cicer arietinum*) [7], soybean (*Glycine max*) [8], rice (*Oryza sativa*) [5], maize (*Zea mays*) [9], and wheat (*Triticum aestivum*) [10]. Like regular QTLs, SDLs can be mapped using DNA markers.

A high-resolution genetic linkage map plays fundamental roles in QTL and association mapping, comparative genomics, map-based cloning, and molecular breeding. One precondition for the construction of high-resolution genetic linkage maps is availability of polymorphic DNA markers. To date, many hexaploid wheat (*Triticum aestivum* L.) linkage maps have been constructed using different types of markers, including restriction fragment length polymorphisms (RFLPs), simple sequence repeats (SSRs), diversity arrays technology (DArT), and single nucleotide polymorphisms (SNPs) [11–21]. SSR markers are informative, and many of them are genome specific, so they are suitable for serving as a framework to anchor other markers in a map. Limitations in the number of available SSRs and in throughput for marker analysis, however, make them unsuitable for high-resolution mapping. SNPs are the most abundant and suitable for high-throughput multiplex analysis and thus are ideal for developing high-density genetic maps for QTL mapping and other genetic analyses [22]. In common wheat, progress in SNP discovery and detection has been slow because of the highly repetitive nature of genome sequences [23]. Most currently available wheat genetic maps have poor marker coverage and were constructed mainly using a single population, so maps may not be useful for QTL analysis and marker-assisted breeding. A high-throughput wheat SNP chip recently was developed by the International Wheat SNP Consortium, and a high-density wheat SNP consensus map of 7,504 SNPs was constructed using seven mapping populations [21]. The SNP chip and map developed from the study provide a powerful resource for further mapping of important traits and for genome-wide association studies in wheat.

The development of a high-density genetic linkage map, especially a consensus map that integrates two or more linkage maps constructed using different populations, facilitates identification of genes for agronomic important traits and whole-genome scanning to identify the common positions and effects of SDLs and understanding of the various mechanisms of segregation distortion. In the present study, we constructed maps using both SNPs and SSRs markers and two wheat RIL populations derived from the crosses of Ning7840 × Clark and Heyne × Lakin and investigated segregation distortion in the maps. The high-density maps developed in this study will be useful for mapping of genes of important wheat traits and developing high-throughput markers for marker-assisted breeding.

## 2. Materials and Methods

### 2.1. Mapping Populations

Two RIL mapping populations were derived from wheat crosses of Ning7840 × Clark and Heyne × Lakin by single-seed descent at Purdue University, West Lafayette, IN, and at Kansas State University, Manhattan, KS, respectively. The population sizes were 127 F_8_ RIL for Ning7840 × Clark and 146 F_6_ RIL for Heyne × Lakin. Ning7840 is a Chinese hard red facultative elite breeding line with the pedigree of (Avrora × Anhui 11) × Sumai 3, and Clark is a soft red winter wheat cultivar developed at Purdue University [24]; Heyne and Lakin are both hard white winter wheat developed at Kansas State [25, 26].

### 2.2. DNA Extraction and Marker Analysis

Genomic DNA isolation and PCRs for SSRs were conducted following the previously described protocols [27]. About 2000 SSR primers from different sources [13–15] (http://wheat.pw.usda.gov/) were used to screen the parents and polymorphic primers were used to analyze the populations. PCR fragments were separated by ABI PRISM 3730 DNA Analyzer (Applied Biosystems, Foster City, CA, USA) and scored using GeneMarker version 1.6 (Soft Genetics LLC, State College, PA, USA).

SNP genotyping was performed using 9 K wheat Infinium iSelect SNP genotyping arrays developed by Illumina Inc. (San Diego, CA). The SNP primers were assembled by the International Wheat SNP Consortium [21]. The SNP assays were conducted at the USDA Small Grains Genotyping Laboratory in Fargo, ND. SNP genotypes were determined using GenomeStudio v2011.1 (Illumina, San Diego CA).

### 2.3. Linkage Map Construction

All SNPs with less than 5% missing data were selected to construct two individual maps and one consensus map using Carthagène software [28]. For each population, both SNP and SSR data were grouped using the “Group” command with a dist-threshold of 0.3 and a LOD-threshold of 10 in Carthagène [28]. Each group was then mapped using the “Nicemapd” command to compute a marker order using two-point distances. The best log-likelihood map was identified using the “Annealing” command that optimized the maximum multipoint log-likelihood using a dedicated simulated annealing stochastic optimization algorithm. The map was further tested using a flips algorithm that checked all possible permutations in a sliding window of fixed size (size ≦ 5) and a polishing algorithm that checked the reliability of the map by removing one marker from the initial map and trying to insert it in all possible intervals. Each group was assigned to 21 wheat chromosomes according to the SSR loci that had known chromosome positions, and the marker orders were checked referring to a publicly available wheat consensus map [15]. Small linkage groups that belonged to the same chromosome were merged together by setting the dist-threshold at 0.5 and the LOD-threshold at 3.0.

The consensus map was constructed by merging the two individual sets of marker data using the “*dsmergen*” command and grouping them using the “*group*” command with the dist-threshold at 0.3 and LOD-threshold at 26. The other steps and parameters were set similar to what was previously described for constructing the individual map. Maps were drawn using MapChart 2.2 [29].

### 2.4. Analysis of Segregation Distortion Loci

The refined datasets of two individual linkage maps were loaded into QTL IciMapping 3.2 [30] to determine SDMs using the SDL module. Goodness of fit to a Mendelian 1 : 1 segregation ratio in both RIL populations was evaluated by Chi-square tests at a 5% significance level. An SDR was declared when three or more closely linked markers exhibited significant segregation distortion.

## 3. Results

### 3.1. Map Construction

Number of markers, marker density, and map length of the three genomes and seven homologous groups are listed in Table 1 and Supplementary Table S1 (in the Supplementary Material available online at http://dx.doi.org/10.1155/2015/830618) for both individual and consensus maps. In the Ning7840 × Clark population, 2770 polymorphic markers (2404 SNPs and 366 SSRs) were used to construct the linkage map, and 2654 (2384 SNPs and 270 SSRs) were mapped. The total map length was 4066.3 cM at an average marker interval of 1.53 cM. Numbers of markers and map lengths were significantly different among the three genomes, with 1152 markers covering 1698.7 cM in A-genome, 1265 markers covering 1687.1 cM in B-genome, and 237 markers covering 680.5 cM in D-genome. The marker densities were 1.47 cM per marker for A-genome, 1.33 for B-genome, and 2.87 for D-genome.

In the Heyne × Lakin population, among 2241 polymorphic markers (2071 SNPs and 170 SSRs) that were used to construct the linkage map, 2173 markers (2050 SNPs and 123 SSRs) were mapped. The map length was 2951.2 cM, with an average marker interval of 1.36 cM across three genomes. Similar to the Ning7840 × Clark map, the A-, B-, and D-genome have 978, 1019, and 176 markers, with map lengths of 1222.7, 1260.8, and 467.7 cM and marker densities of 1.25, 1.24, and 2.66 cM/marker for each genome, respectively. The variations in the three parameters among the seven homologous groups were relatively smaller than those among genomes in both maps. In the Ning7840 × Clark map, the map lengths and marker densities of different homologous groups varied from 374.4 cM at 1.11 cM/marker (Group 1) to 764.4 cM at 2.03 cM/marker (Group 7), whereas in the Heyne × Lakin map, the map length and marker density ranged from 286.7 cM at 0.85 cM/marker (Group 6) to 602.1 cM at 1.52 cM/marker (Group 3).

The consensus map was built by merging the two sets of marker data together from the two mapping populations. Among 4200 makers, including the 3714 SNPs and 486 SSRs used to construct the consensus map, only 811 markers overlapped between the two maps. Consequently, 3686 markers (88%), 3543 SNPs, and 143 SSRs were assembled into 36 linkage groups, representing all 21 wheat chromosomes. The map covered 3258.7 cM in genetic distance at an average density of 0.88 cM/marker. The A-, B-, and D-genome contained 1684, 1744, and 258 markers at map lengths of 1384.4, 1430.5, and 443.8 cM and marker density of 0.82, 0.82, and 1.72 cM/marker, respectively. Among the seven homologous groups, the map lengths ranged from 357.2 (Group 4) to 638.1 cM (Group 7), and the marker densities were from 0.63 (Group 6) to 1.24 cM/marker (Group 7). A total of 3679 loci were mapped to a single chromosome with only seven SNPs that could be mapped to two chromosomes in the same homologous group; for example,* IWA2861* and* IWA5076* were mapped on both chromosomes 1A and 1B,* IWA5685* was mapped on both 2A and 2D, and* IWA766*,* IWA6994*,* IWA1786*, and* IWA7066* were mapped on both 5B and 5D. Thus, most mapped markers are chromosome-specific in the consensus map.

### 3.2. Segregation Distortion in Two Maps

Among the 2654 mapped markers in the Ning7840 × Clark map, 490 (18.5%) showed significant segregation distortion at *P* < 0.05 (Table 2). Most of these SDMs (427) had predominant alleles from Clark, and only about 13% (63) of SDMs showed bias toward Ning7840 alleles. The number of SDMs was unevenly distributed over different chromosomes, ranging from none (4D) to 152 (6B) and from 27 (Group 4) to 165 (Group 6) among homologous groups. The B-genome had the most SDMs (287), and the D-genome had the fewest ones (25). About 57% of SDMs were from three chromosomes, 1B (57), 6B (152), and 7A (69). Thirty significant SDRs (*P* < 0.05) were located on 12 chromosomes (1A, 1B, 2A, 2B, 3A, 4B, 5A, 5B, 6A, 6B, 7A, and 7D) (Table 3). All of the SDMs in a given SDR skewed toward the same parent; in most cases, this was the male parent Clark, except for three SDRs on 4B and 7A that distorted toward the female parent Ning7840.

Fewer SDMs (10.4% of 2173 markers) were mapped in the Heyne × Lakin than in Ning7840 × Clark map. Most of the SDMs (158) had predominant alleles from Lakin, and 67 SDMs showed bias toward Heyne. Most of the SDMs were located on chromosomes 2B (74) and 3B (58). Similar to the Ning7840 × Clark map, B-genome had the most SDMs and D-genome the fewest ones, and homologous Group 2 had the most SDMs (86) and Group 4 the fewest ones (3). A total of 11 SDRs were found on seven chromosomes (2A, 2B, 2D, 3B, 5B, 5D, and 7A) at* P* < 5%. Five SDRs on chromosomes 2D, 3B, 5B, and 7A distorted toward male parent Lakin, whereas four SDRs on chromosomes 2A and 5D distorted toward female parent Heyne. On chromosome 2B, each SDR distorted toward Lakin and Heyne, respectively (Table 3).

Most SDRs were located on different chromosome locations in the two maps. Only one SDR on chromosome 2B completely overlapped in the two maps. Two SDLs in the marker intervals of* IWA8195-IWA4890* and* Xgwm3.2-Xgwm47* on chromosome 2B of the Ning7840 × Clark map can be located in the SDR interval of* IWA5810-Xgwm120* in the Heyne × Lakin map. The SDR showed segregation distortion toward the male parents in both maps.

## 4. Discussion

### 4.1. Mapping Populations

In this study, two wheat RIL populations were used to construct the genetic maps. In the Ning7840 × Clark population, the two parents are genetically and geographically far from each other, because Ning7840 is a hard facultative wheat from China and Clark is a soft winter wheat from the US [24]. The parents exhibit significant agronomic differences, including adaptation and yield-related traits, end-use quality traits, and multiple disease resistance [31, 32]; for example, Ning7840 is resistant to rusts and* Fusarium* head blight but susceptible to Hessian fly [33], whereas Clark has opposite reactions to these pests. Clark also adapts well to US growing environments, but Ning7840 does not. Thus, a high-density map is essential for mapping these traits and identification of markers linked to QTLs underlining these traits for marker-assisted breeding.

Marza et al. [32] previously used this population to construct a genetic map with 363 amplified fragment length polymorphism (AFLP) markers and 47 SSR markers and mapped some of these traits; however, no sequence information is available for AFLP markers, so these markers that link to certain QTLs cannot be converted to breeder-friendly markers for breeding application. Also, the 47 SSR markers used in the previous study did not cover all 21 chromosomes to serve as a framework for the map. In this study, we mapped 2404 SNPs and 366 SSRs in the map, about six times more markers than in the previous map. These SNP markers all have sequence information that can be used to develop breeder-friendly markers. This map should be very useful in mapping QTLs for contrasting traits between Ning7840 and Clark and in identifying closely linked markers for marker-assisted selection of these QTLs.

In the Heyne × Lakin population, as expected, fewer polymorphic markers (2071 SNPs and 170 SSRs) were mapped than for the Ning7840 × Clark population because Heyne and Lakin have a closer genetic relationship and were released from breeding programs in Kansas. Heyne and Lakin are both hard white winter wheat and differ in resistance to leaf, stem, and stripe rusts and to wheat spindle streak virus, speckled leaf blotch, tan spot, powdery mildew, and* Fusarium* head blight, with resistance genes mainly from Heyne (http://www.ksre.ksu.edu/bookstore/pubs/l922.pdf) [25, 34]. Lakin has good noodle quality with a low level of polyphenol oxidase (PPO), which is a very important end-use quality trait for noodle production. This map will be useful in identifying high-throughput SNP markers linked to QTLs for local sources of resistance to multiple diseases and for characterization of QTLs for noodle-making qualities in white wheat.

### 4.2. Genetic Maps for Winter Wheat

Several types of markers have been used for map construction [11, 12, 15, 17, 23, 32], but most of those maps either have low resolution or consist of markers that are unsuitable for high-throughput screening of breeding materials. SNP markers are practically unlimited in number, and many high-throughput SNP detection systems have been developed for routine analysis, so SNP has become a popular type of marker in development of high-density genetic maps. In wheat, the first SNP-based map was constructed with 480 SNPs and 574 non-SNP markers covering 2999 cM in distance and with marker density of 2.8 cM per marker [18]. More recently, the International Wheat SNP Consortium assembled the wheat 9 K SNP chip and built the first high-density SNP-based consensus map using seven mapping populations [21]. This work mapped 7504 SNP markers, covering 4740.5 cM with a marker density of 1.9 SNP/cM. This SNP map is a very useful source of SNP for map construction and high-resolution QTL mapping in wheat. In the current study, we constructed a consensus map with 3541 SNPs that covered 3258.7 cM with an average marker density of 0.88 marker/cM. We also mapped 145 SSRs as a framework in the consensus map to anchor SNP to each chromosome, so this map provides connections between SNP and SSR markers and can be used for comparative mapping of QTLs linked to SSR markers previously reported in different studies. Although the number of SNPs mapped in the current study was about half that of Cavanagh et al. [21], the studies shared a total of 3415 SNPs [21], and the chromosomal locations and relative genetic distance of most SNPs were consistent, with only 145 SNPs mapped on different chromosomes (data not shown). Among the markers with inconsistent chromosome locations, 42 located on 2A in our map were located on chromosome 5B of Cavanagh's map [21]. In the consensus map, some gaps in one individual map can be filled with markers from another map, indicating that a marker that is monomorphic in one mapping population can be polymorphic in another population. Thus, constructing a consensus map by combining the data from different populations can help saturate a map and improve map resolution. The current wheat consensus map also integrated both SNP and SSR markers in the map and both populations segregated for many important traits, so the map should be very useful for fine-mapping and map-based cloning of QTLs for these traits.

### 4.3. Segregation Distortion

Segregation distortion is common in plants and has been reported in many species [6]. It might be a powerful force in the evolution of many fundamental aspects of sexual reproduction [35]. Biological segregation distortion is always associated with a cluster of markers (SDMs) within a chromosomal region that harbors the SDL, so most SDMs in the region are clustered together to form SDR. This phenomenon has been reported in several crops, including wheat [36], rice [37], maize [38], and barley [6]. This study agrees with the observations from previous studies. In general, SDR was declared when three or more closely linked markers exhibited significant segregation distortion [6]. In this study, 379 SDMs were clustered in 30 SDRs with 3 to 135 SDMs per SDR in the Ning7840 × Clark population, and 186 SDMs were grouped in 11 SDRs with 3 to 46 SDMs in the Heyne × Lakin population. The higher number of SDRs and SDMs in Ning7840 × Clark than in Heyne × Lakin may be partially explained by the fact that Ning7840 is genetically and geographically far from Clark, whereas Heyne and Lakin are more closely related. Thus, the level of genetic difference between parents may be one of the causes of difference in number of SDRs between the two populations in this study. Translocation of alien fragments into wheat is another source of segregation distortion.* Sr36* for stem rust resistance and* Pm6* for powdery mildew resistance on wheat chromosome 2B from the short arm of chromosome 2G of* T. timopheevii *are preferentially transmitted in different backgrounds and have obvious segregation distortion [39–41]; the SNP map from multiple populations also supports this observation [21]. However,* Sr40* for stem rust resistance on 2B chromosome transferred from 2G of* T. timopheevii* showed segregation distortion in one background but not the other [42], so the presence of SDRs may vary with sources and backgrounds of transferred alien fragments, which is supported by the results from the current study. Pedigree analysis indicated that both Ning7840 and Heyne have an alien chromosome translocation with 1BS.1RS from rye in Ning7840 (NGRP 2005) and short 2NS.2AS fragment translocation from* Triticum ventricosum* in Heyne. In Ning7840 × Clark, 1BS/1RS arm translocation contributed an SDR, but 2NS in Heyne did not cause significant segregation distortion in chromosome 2A (Table 3), which suggests that not all alien fragment translocations lead to SDR, but if this occurs, segregation usually distorted toward the parent without an alien fragment, such as Clark in the Ning7840 × Clark population. However, in the Ning7840 × Clark map, SDMs were located on several chromosomes, including 1B, 2B, 3A, 6B, 7A, and 7D, with most SDMs on 1B and 6B (Table 3). The largest SDR with the most SDMs is on chromosome 6B. In the Heyne × Lakin map, most SDMs are on chromosomes 2B, 3B, and 5B (Table 3). The segregation mainly distorted toward male parents in both maps. These SDRs cannot be directly explained by alien fragment translocation; therefore, some other mechanisms, including preferential pollination, may be involved in segregation distortion in wheat RIL population.

**Figure 1:**
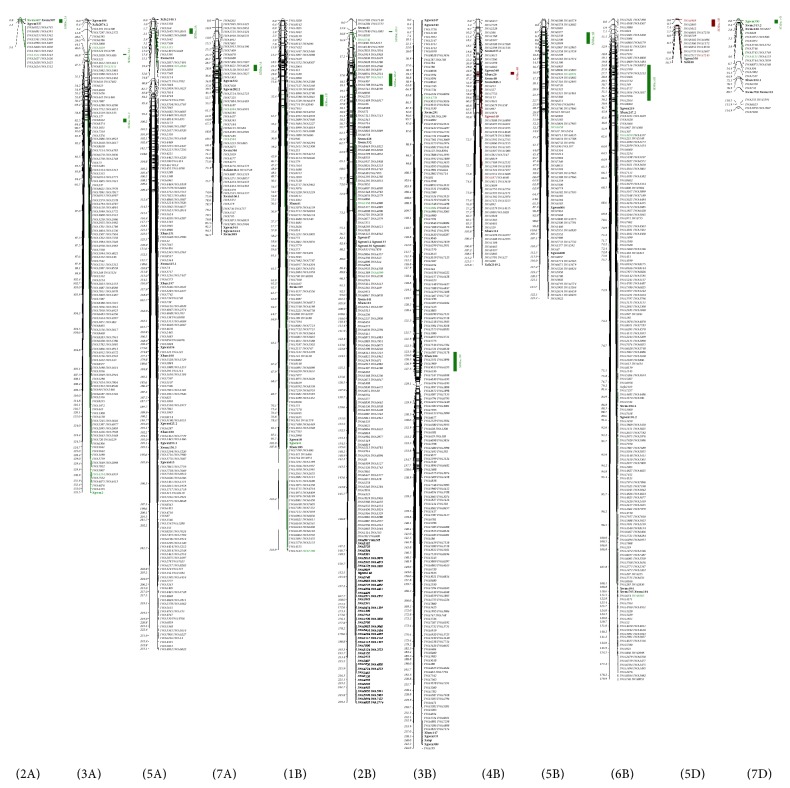
Segregation distorted region (SDR) detected in the consensus map. The majority of the distorted markers clustered in the SDRs on chromosomes 1B, 2A, 2B, 3A, 3B, 4B, 5A, 5B, 5D, 6B, 7A, and 7D in the consensus map. SSR markers are identified in bold type, and SDRs are labeled with green or brown, indicating SDR bias to the male or female parent, respectively.

In the consensus map, 630 SDMs were mapped, and mainly clustered to 14 SDRs on chromosomes 1B, 2A, 2B, 3A, 3B, 4B, 5A, 5B, 5D, 6B, 7A, and 7D (Figure 1). Most SDRs skewed toward male parents, except SDRs on 4B and 5D, which skewed toward female parents. These results suggest that SDRs are real in RIL populations and that SDMs constitute 17.1% of markers mapped in this study, so they cannot be neglected in QTL mapping. One concern is that segregation distortion may affect the recombination frequencies and thus impede mapping precision in map construction and QTL detection. The common practice in QTL mapping is using SDMs to construct a linkage map using Mendelian marker loci first and then inserting distorted markers into the map one by one to fill the missing portion of the genome. In many map construction cases, however, distorted markers are simply removed for map construction and QTL analysis, so all QTLs in SDRs are not mapped in these cases. Doucleff et al. [43] suggested that SDMs detected at the 1% level rather than the 5% level should be excluded in mapping to reduce the frequency of false positives. If distortion is not extremely serious, the effect from distortion actually can be trivial in a large mapping population. Zhang et al. [5] demonstrated that segregation distortion may not necessarily increase number of false QTLs or significantly affect the estimation of QTL positions and effects. Xu [2] also showed that the presence of SDLs was not necessarily detrimental to QTL mapping because SDLs can either decrease or increase the statistical power of QTL mapping; indeed, many studies have shown important QTL located in highly distorted chromosome regions. For example, Li et al. [44] identified major QTL conferring resistance to crown rot in barley that was located in one of the SDRs on chromosome 3H, and Bovill et al. [45] reported an association between SDRs and QTLs or genes for crown rot resistance in wheat. The* Sr36* locus was in the SDR on chromosome 2B [40–42]. In the present study, an SDR identified in both individual maps was located near the marker* Xgwm47* on 2B, where several rust resistance loci, including* Yr7*,* Sr9a,* and* Lr50,* were mapped (Wheat-Composite 2004-2B map in GrainGenes: http://wheat.pw.usda.gov/). Torp et al. [46] also identified QTL (*QGpp.kvl-2B.1*) in this chromosomal region that affects embryo formation and plant regeneration in double haploid population and also results in segregation distortion in the region surrounding the QTL. In this study, large variation among SDRs was observed (Table 3), with some having a large number of SDMs and covering a long chromosome fragment and others having only one or a few markers covering a very short genetic distance. Some SDRs in the latter case might be the result of genotyping error and could be removed for map construction, but a large SDR with many markers should be included in the map with special care. A large population may facilitate creation of desired recombinants for QTL mapping and breeding selection.

## 5. Conclusion

A high-density consensus map was constructed using SNP and SSR markers by merging two genetic maps developed from two RIL populations, Ning7840 × Clark and Heyne × Lakin. A total of 3541 SNPs and 145 SSRs were mapped, and the map covered 3258.7 cM in genetic distance with an average interval of 0.88 cM. These high-density maps can be useful for mapping QTLs of many important wheat traits because their parents have significant contrasts in many of these traits. Chromosome regions with obvious segregation distortion were identified in these maps. Approximately 19% of mapped markers showed distorted segregation in the Ning7840 × Clark population and 10.4% in the Heyne × Lakin population. The mapped distorted makers (630) in the consensus map accounted for 17.1% of total markers in the map. The majority of the distorted markers clustered in the SDRs on 12 chromosomes. All of the markers in a given SDR skewed toward one of the parents, suggesting that gametophytic competition during zygote formation was likely one of the causes for segregation distortion in the populations.

## Supplementary Material

Marker names and genetic distance in cM between markers for the two individual maps and their consensus map constructed from Ning7840 × Clark and Heyne × Lakin populations.

## Figures and Tables

**Table 1:** Description of the two individual maps and their consensus map constructed from Ning7840 × Clark and Heyne × Lakin populations.

Chr.	Ning × Clark map			Heyne × Lakin map			Consensus map		
	NM	LGL	MD	NM	LGL	MD	NM	LGL	MD
1A	125	204.0	1.63	110	169.2	1.54	188	177.1	0.94
1B	181	108.8	0.60	98	107.5	1.10	227	116.9	0.51
1D	30	61.6	2.05	44	103.6	2.35	49	89.5	1.83
Group 1	336	374.4	1.11	252	380.3	1.51	464	383.5	0.83

2A	103	175.1	1.70	134	170.5	1.27	161	98.1	0.61
2B	218	346.8	1.59	161	198	1.23	302	278.2	0.92
2D	63	154.0	2.44	28	80.6	2.88	61	128.2	2.10
Group 2	384	675.9	1.76	323	449.1	1.39	524	504.5	0.96

3A	186	285.7	1.54	124	185.8	1.50	265	242.7	0.92
3B	156	269.5	1.73	257	305	1.19	305	244	0.80
3D	26	91.0	3.50	15	111.3	7.42	18	11.2	0.62
Group 3	368	646.2	1.76	396	602.1	1.52	588	497.9	0.85

4A	129	204.8	1.59	189	163.3	0.86	249	208.6	0.84
4B	85	158.6	1.87	29	98.5	3.40	93	125.8	1.35
4D	6	19.8	3.30	9	35.2	3.91	8	22.8	2.85
Group 4	220	383.2	1.74	227	297	1.31	350	357.2	1.02

5A	173	271.4	1.57	158	208	1.32	256	233.1	0.91
5B	310	347.4	1.12	164	276.4	1.69	337	231.3	0.69
5D	25	54.0	2.08	24	18.1	0.75	29	21.1	0.73
Group 5	508	672.8	1.32	346	502.5	1.45	622	485.5	0.78

6A	209	207.6	0.99	105	118.2	1.13	273	156	0.57
6B	204	202.2	0.99	195	139.6	0.72	289	179.9	0.62
6D	48	139.6	2.91	36	28.9	0.80	60	56.1	0.94
Group 6	461	549.4	1.19	336	286.7	0.85	622	392.0	0.63

7A	227	350.1	1.54	148	205.9	1.39	292	268.8	0.92
7B	111	253.8	2.29	125	141.9	1.14	191	254.4	1.33
7D	39	160.5	4.12	20	90	4.50	33	114.9	3.48
Group 7	377	764.4	2.03	293	437.8	1.49	516	638.1	1.24

A-genome	1152	1698.7	1.47	978	1222.7	1.25	1684	1384.4	0.82
B-genome	1265	1687.1	1.33	1019	1260.8	1.24	1744	1430.5	0.82
D-genome	237	680.5	2.87	176	467.7	2.66	258	443.8	1.72
Whole genome	2654	4066.3	1.53	2173	2951.2	1.36	3686	3258.7	0.88

NM: number of markers mapped on each chromosome.

MD: average marker density in cM per marker.

LGL: linkage group length in cM.

**Table 2:** Chromosome distributions of markers that show segregation distortion (*P* < 0.05).

Chr.	Ning7840 × Clark map			Heyne × Lakin map		
	Number of distorted markers	Distortion toward Ning7840	Distortion toward Clark	Number of distorted markers	Distortion toward Heyne	Distortion toward Lakin
1A	17	6	11	2	2	0
1B	57	0	57	0	0	0
1D	1	1	0	5	4	1
Group 1	75	7	68	7	6	1

2A	30	0	30	3	3	0
2B	28	1	27	74	26	48
2D	2	1	1	9	0	9
Group 2	60	2	58	86	29	57

3A	31	3	28	4	3	1
3B	9	5	4	58	0	58
3D	1	0	1	1	0	1
Group 3	41	8	33	63	3	60

4A	2	2	0	2	1	1
4B	25	25	0	1	0	1
4D	0	0	0	0	0	0
Group 4	27	27	0	3	1	2

5A	19	4	15	0	0	0
5B	9	1	8	28	1	27
5D	1	0	1	20	20	0
Group 5	29	5	24	48	21	27

6A	10	0	10	1	1	0
6B	152	1	151	7	3	4
6D	3	1	2	1	0	1
Group 6	165	2	163	9	4	5

7A	69	10	59	9	3	6
7B	7	2	5	0	0	0
7D	17	0	17	0	0	0
Group 7	93	0	17	9	3	6

A-genome	178	25	153	21	13	8
B-genome	287	35	252	168	30	138
D-genome	25	3	22	36	24	12
Total	490	63	427	225	67	158

**Table 3:** SDRs detected in the maps of Ning7840 × Clark and Heyne × Lakin at *P* < 0.05.

SDR	Chr.	Number of markers	Genetic distance (cM)	Distortion toward parent
Ning7840 × Clark map				
* IWA4506-IWA1481*	1A	5	11.2	Clark
* IWA5352-IWA2064*	1B	3	2.2	Clark
* IWA7037-IWA1859*	1B	4	6.9	Clark
* IWA406-Xbarc152*	1B	40	8.5	Clark
* Xgwm537-IWA3382*	2A	20	5.4	Clark
* IWA5244-IWA7864*	2A	10	11	Clark
* IWA8195-IWA4890*	2B	3	5.7	Clark
* Xgwm3.2-Xgwm47*	2B	4	0.4	Clark
* IWA10-IWA5240*	2B	5	2.9	Clark
* IWA2008-IWA3111*	3A	10	3.7	Clark
* IWA4451-IWA7712*	3A	5	0	Clark
* IWA3739-Xgwm2*	3A	9	7.6	Clark
* Xgwm149-IWA187*	4B	19	3.9	Ning7840
* Xbarc163-IWA329*	4B	4	10.3	Ning7840
* IWA2840-IWA3335*	5A	10	6.8	Clark
* IWA6024-IWA3226*	5B	3	0	Clark
* IWA5398-IWA7764*	6A	9	1.2	Clark
* IWA5857-Xwmc705*	6B	5	19.4	Clark
* Xbarc185.3-Xbarc1033*	6B	135	43.6	Clark
* IWA1629-IWA3221*	6B	3	0	Clark
* IWA5204-IWA1741*	6B	6	4.9	Clark
* IWA2012-IWA1111*	7A	19	17.1	Clark
* IWA8066-IWA204*	7A	13	0	Clark
* IWA6261-IWA1424*	7A	5	0	Ning7840
* IWA5913-IWA5167*	7A	9	5.4	Clark
* IWA4994-IWA8204*	7A	6	4.4	Clark
* IWA828-IWA827*	7D	5	0.4	Clark
* IWA4131-IWA3745*	7D	3	0	Clark
* Xwmc313.2-Xwmc313.4*	7D	3	6.3	Clark
* Xbarc252.1-Xwmc222*	7D	4	8.1	Clark
Heyne × Lakin map				
* IWA6798-IWA2601*	2A	3	2.1	Heyne
* IWA5810-Xgwm120*	2B	46	29	Lakin
* IWA2115-IWA7370*	2B	26	38.9	Heyne
* IWA6302-Xcfd51*	2D	9	15.1	Lakin
* IWA3901-IWA4498*	3B	38	4.2	Lakin
* Xbarc344-IWA4778*	3B	12	16.3	Lakin
* IWA8391-IWA7966*	5B	26	38.5	Lakin
* IWA4969-IWA6001*	5D	5	7.9	Heyne
* IWA8102-IWA2607*	5D	8	0.7	Heyne
* IWA1428-Xgwm272*	5D	7	4.2	Heyne
* IWA4594-IWA84*	7A	6	0.3	Lakin

## References

[1] Lyttle T. W. (1991). Segregation distorters. *Annual Review of Genetics*.

[2] Xu S. Z. (2008). Quantitative trait locus mapping can benefit from segregation distortion. *Genetics*.

[3] Lorieux M., Goffinet B., Perrier X., de León D. G., Lanaud C. (1995). Maximum-likelihood models for mapping genetic markers showing segregation distortion. 1. Backcross populations. *Theoretical and Applied Genetics*.

[4] Lorieux M., Perrier X., Goffinet B., Lanaud C., de León D. G. (1995). Maximum-likelihood models for mapping genetic markers showing segregation distortion. 2. F_2_ populations. *Theoretical and Applied Genetics*.

[5] Zhang L. Y., Wang S. Q., Li H. H. (2010). Effects of missing marker and segregation distortion on QTL mapping in F_2_ populations. *Theoretical and Applied Genetics*.

[6] Li H. B., Kilian A., Zhou M. X. (2010). Construction of a high-density composite map and comparative mapping of segregation distortion regions in barley. *Molecular Genetics and Genomics*.

[7] Castro P., Rubio J., Cabrera A., Millán T., Gil J. (2011). A segregation distortion locus located on linkage group 4 of the chickpea genetic map. *Euphytica*.

[8] Baumbach J., Rogers J. P., Slattery R. A. (2012). Segregation distortion in a region containing a male-sterility, female-sterility locus in soybean. *Plant Science*.

[9] Wang G., He Q. Q., Xu Z. K., Song R. T. (2012). High segregation distortion in maize B73 x teosinte crosses. *Genetics and Molecular Research*.

[10] Takumi S., Motomura Y., Iehisa J. C. M., Kobayashi F. (2013). Segregation distortion caused by weak hybrid necrosis in recombinant inbred lines of common wheat. *Genetica*.

[11] Liu Y.-G., Tsunewaki K. (1991). Restriction fragment length polymorphism (RFLP) analysis in wheat. II. Linkage maps of the RFLP sites in common wheat. *Japanese Journal of Genetics*.

[12] Anderson J. A., Ogihara Y., Sorrells M. E., Tanksley S. D. (1992). Development of a chromosomal arm map for wheat based on RFLP markers. *Theoretical and Applied Genetics*.

[13] Röder M. S., Korzun V., Wendehake K. (1998). A microsatellite map of wheat. *Genetics*.

[14] Paillard S., Schnurbusch T., Winzeler M. (2003). An integrative genetic linkage map of winter wheat (*Triticum aestivum* L.). *Theoretical and Applied Genetics*.

[15] Somers D. J., Isaac P., Edwards K. (2004). A high-density microsatellite consensus map for bread wheat (*Triticum aestivum* L.). *Theoretical and Applied Genetics*.

[16] Liu Z. H., Anderson J. A., Hu J., Friesen T. L., Rasmussen J. B., Faris J. D. (2005). A wheat intervarietal genetic linkage map based on microsatellite and target region amplified polymorphism markers and its utility for detecting quantitative trait loci. *Theoretical and Applied Genetics*.

[17] Francki M. G., Walker E., Crawford A. C. (2009). Comparison of genetic and cytogenetic maps of hexaploid wheat (*Triticum aestivum* L.) using SSR and DArT markers. *Molecular Genetics and Genomics*.

[18] Allen A. M., Barker G. L. A., Berry S. T. (2011). Transcript-specific, single-nucleotide polymorphism discovery and linkage analysis in hexaploid bread wheat (*Triticum aestivum* L.). *Plant Biotechnology Journal*.

[19] Poland J. A., Brown P. J., Sorrells M. E., Jannink J.-L. (2012). Development of high-density genetic maps for barley and wheat using a novel two-enzyme genotyping-by-sequencing approach. *PLoS ONE*.

[20] Huang B. E., George A. W., Forrest K. L. (2012). A multiparent advanced generation inter-cross population for genetic analysis in wheat. *Plant Biotechnology Journal*.

[21] Cavanagh C. R., Chao S., Wang S. (2013). Genome-wide comparative diversity uncovers multiple targets of selection for improvement in hexaploid wheat landraces and cultivars. *Proceedings of the National Academy of Sciences of the United States of America*.

[22] Rafalski A. (2002). Applications of single nucleotide polymorphisms in crop genetics. *Current Opinion in Plant Biology*.

[23] Ganal M. W., Röder M. S., Varshney P. K., Tuberosa R. (2007). Microsatellite and SNP markers in wheat breeding. *Genomics-Assisted Crop Improvement: Vol 2: Genomics Applications in Crops*.

[24] Ohm H. W., Shaner G., Foster J. E., Patterson F. L., Buechley G. (1988). Registration of ‘Clark’ wheat. *Crop Science*.

[25] Sears R. G., Martin T. J., McCluskey P. J. (2001). Registrations of Heyne cultivar. *Crop Science*.

[26] Martin J. T., Fritz A., Shroyer J. P. (2001). *Lakin Hard White Wheat*.

[27] Zhang D. D., Bai G. H., Zhu C. S., Yu J. M., Carver B. F. (2010). Genetic diversity, population structure, and linkage disequilibrium in U.S. elite winter wheat. *The Plant Genome*.

[28] Schiex T., de Givry S., Chabrier P., Bouchez M., Nelson C. https://mulcyber.toulouse.inra.fr/projects/carthagene/.

[29] Voorrips R. E. (2002). Mapchart: software for the graphical presentation of linkage maps and QTLs. *Journal of Heredity*.

[30] Wang J. K., Li H. H., Zhang L. Y., Meng L. (2013). *Users' Manual of QTL IciMapping Version 3.3*.

[31] Bai G. H., Kolb F. L., Shaner G. E., Domier L. L. (1999). Amplified fragment length polymorphism markers linked to a major quantitative trait locus controlling scab resistance in wheat. *Phytopathology*.

[32] Marza F., Bai G. H., Carver B. F., Zhou W. C. (2006). QTL for yield and related traits in the wheat population Ning7840 x Clark. *Theoretical and Applied Genetics*.

[33] Li C. L., Chen M. S., Chao S. M., Yu J. M., Bai G. H. (2013). Identification of a novel gene, *H34*, in wheat using recombinant inbred lines and single nucleotide polymorphism markers. *Theoretical and Applied Genetics*.

[34] Zhang X. H., Bai G. H., Bockus W., Ji X. J., Pan H. Y. (2012). Quantitative trait loci for *Fusarium* head blight resistance in U.S. hard winter wheat cultivar Heyne. *Crop Science*.

[35] Taylor D. R., Ingvarsson P. K. (2003). Common features of segregation distortion in plants and animals. *Genetica*.

[36] Alheit K. V., Reif J. C., Maurer H. P. (2011). Detection of segregation distortion loci in triticale (x *Triticosecale* Wittmack) based on a high-density DArT marker consensus genetic linkage map. *BMC Genomics*.

[37] Xu Y., Zhu L., Xiao J., Huang N., McCouch S. R. (1997). Chromosomal regions associated with segregation distortion of molecular markers in F_2_, backcross, doubled haploid, and recombinant inbred populations in rice (*Oryza sativa* L.). *Molecular and General Genetics*.

[38] Lu H., Romero-Severson J., Bernardo R. (2002). Chromosomal regions associated with segregation distortion in maize. *Theoretical and Applied Genetics*.

[39] Allard R. W., Shands R. G. (1954). Inheritance of resistance to stem rust and powdery mildew in cytologically stable spring wheats derived from *Triticum timopheevi*. *Phytopathology*.

[40] Brown-Guedira G. L., Singh S., Fritz A. K. (2003). Performance and mapping of leaf rust resistance transferred to wheat from *Triticum timopheevii* subsp. *armeniacum*. *Phytopathology*.

[41] Tsilo T. J., Jin Y., Anderson J. A. (2008). Diagnostic microsatellite markers for the detection of stem rust resistance gene *Sr36* in diverse genetic backgrounds of wheat. *Crop Science*.

[42] Wu S. Y., Pumphrey M., Bai G. (2009). Molecular mapping of stem-rust-resistance gene *Sr40* in wheat. *Crop Science*.

[43] Doucleff M., Jin Y., Gao F., Riaz S., Krivanek A. F., Walker M. A. (2004). A genetic linkage map of grape, utilizing *Vitis rupestris* and *Vitis arizonica*. *Theoretical and Applied Genetics*.

[44] Li H. B., Zhou M. X., Liu C. J. (2009). A major QTL conferring crown rot resistance in barley and its association with plant height. *Theoretical and Applied Genetics*.

[45] Bovill W. D., Ma W., Ritter K. (2006). Identification of novel QTL for resistance to crown rot in the doubled haploid wheat population ‘W21MMT70’ x ‘Mendos’. *Plant Breeding*.

[46] Torp A. M., Hansen A. L., Andersen S. B. (2001). Chromosomal regions associated with green plant regeneration in wheat (*Triticum aestivum* L.) anther culture. *Euphytica*.

